# Toll-like receptor 4 and Syk kinase shape dendritic cell-induced immune activation to major house dust mite allergens

**DOI:** 10.3389/fmed.2023.1105538

**Published:** 2023-08-08

**Authors:** Stefanie Busold, Jaap H. Akkerdaas, Esther M. Zijlstra-Willems, Kees van der Graaf, Sander W. Tas, Esther C. de Jong, Ronald van Ree, Teunis B. H. Geijtenbeek

**Affiliations:** ^1^Amsterdam University Medical Centers, location AMC, Department of Experimental Immunology, Amsterdam, Netherlands; ^2^Amsterdam Institute for Infection and Immunity, Inflammatory Diseases, Amsterdam, Netherlands; ^3^Citeq Biologics, Groningen, Netherlands; ^4^Amsterdam Rheumatology and Immunology Center, Department of Rheumatology and Clinical Immunology, Amsterdam University Medical Centers, University of Amsterdam, Amsterdam, Netherlands; ^5^Amsterdam University Medical Centers, location AMC, Department of Otorhinolaryngology, Amsterdam, Netherlands

**Keywords:** house dust mite allergy, Der p 1, Der p 2, TLR4, Syk, dendritic cell activation

## Abstract

**Background:**

House dust mite (HDM) is a major cause of respiratory allergic diseases. Dendritic cells (DCs) play a central role in orchestrating adaptive allergic immune responses. However, it remains unclear how DCs become activated by HDM. Biochemical functions of the major HDM allergens Der p 1 (cysteine protease) and Der p 2 (MD2-mimick) have been implicated to contribute to DC activation.

**Methods:**

We investigated the immune activating potential of HDM extract and its major allergens Der p 1 and Der p 2 using monocyte-derived DCs (moDCs). Maturation and activation markers were monitored by flow cytometry and cytokine production by ELISA. Allergen depletion and proteinase K digestion were used to investigate the involvement of proteins, and in particular of the major allergens. Inhibitors of spleen tyrosine kinase (Syk), Toll-like receptor 4 (TLR4) and of C-type lectin receptors (CLRs) were used to identify the involved receptors. The contribution of endotoxins in moDC activation was assessed by their removal from HDM extract.

**Results:**

HDM extract induced DC maturation and cytokine responses in contrast to the natural purified major allergens Der p 1 and Der p 2. Proteinase K digestion and removal of Der p 1 or Der p 2 did not alter the immune stimulatory capacity of HDM extract. Antibodies against the CLRs Dectin-1, Dectin-2, and DC-SIGN did not affect cytokine responses. In contrast, Syk inhibition partially reduced IL-6, IL-12 and completely blocked IL-10. Blocking TLR4 signaling reduced the HDM-induced IL-10 and IL-12p70 induction, but not IL-6, while endotoxin removal potently abolished the induced cytokine response.

**Conclusion:**

Our data strongly suggest that HDM-induced DC activation is neither dependent on Der p 1 nor Der p 2, but depend on Syk and TLR4 activation, which might suggest a crosstalk between Syk and TLR4 pathways. Our data highlight that endotoxins play a potent role in immune responses targeting HDM.

## Introduction

Allergic sensitization manifests as a T helper cell type 2 (Th2) response that results in the formation of allergen-specific immunoglobulin E (IgE) antibodies. With 10–30% of the population suffering from allergic rhinitis, airborne allergies contribute to a global public health problem (1). Focusing on the European continent, half of the allergic patients are sensitized toward house dust mites (HDM) (2), which identifies HDM as a main environmental allergen source. More than 30 allergens derived from HDM have been characterized, of which Der p 1 and Der p 2 are recognized by the vast majority of HDM-allergic patients and are hence considered serodominant (3). Those proteins are among the 50 most abundant proteins expressed by HDM (4). However, during natural HDM encounter those major allergens are co-delivered with a spectrum of non-allergenic bystander substances of environmental and microbial nature. While the major HDM allergens harbor the major epitopes recognized by IgE, their role in the initial events of allergenic sensitization is not fully elucidated yet. Dendritic cells (DCs) are important antigen-presenting cells (APC) and as such have a unique role in bridging innate with adaptive immune responses. Being equipped with a broad repertoire of pathogen-sensing receptors, DCs continuously sample their environment for invading pathogens. Their usual sub-epithelial localization positions DCs at the frontline to encounter foreign structures. DCs are important in activating antigen-specific T and B cell responses and shaping immunity. For other allergies it has been shown that the respective purified allergens are poorly immunogenic by themselves (5, 6). However, several major HDM allergens possess biological activities. Der p 1 is a cysteine protease within the mite’s digestive tract (2, 3) and its enzymatic activity might possess immune-activating properties. Several studies reviewed by Zhang et al. have emphasized the biological properties of Der p 1 on epithelial cells (ECs) (7). Besides its possible, yet disputed association with the activation of protease-activated receptors on ECs, the enzymatic activity of Der p 1 has been demonstrated to lead to cleavage of tight junctions increasing epithelial permeability and triggering cytokine release (7–12). In contrast, Der p 2, whose trans-epithelial passage might benefit from the Der p 1-mediated modifications (2, 7), shows structural and functional homology with myeloid differentiation protein-2 (MD-2) and promotes lipopolysaccharide (LPS)-induced signaling via Toll-like receptor 4 (TLR4) (13, 14). This provides Der p 2 with potent auto-adjuvant properties. However, whether the major HDM allergens themselves or rather other substances co-presented in the natural mite environment trigger immune activation via DCs remains unresolved. We here investigated the impact of HDM extract and its major allergens Der p 1 and Der p 2 on DCs as well as the involved pathways of immunogenicity. Our data strongly suggest that proteins, including the major allergens, are not directly involved in DC-mediated immune activation toward HDM. Instead, our data highlight the involvement of TLR4 and spleen tyrosine kinase (Syk) in inducing HDM-directed immune responses via DCs. Further research will show whether TLR4 and Syk are involved in allergic responses and targeting these pathways might attenuate severe allergic reactions.

## Methods

### Allergens and extracts

Whole culture (HDM extract) and mite body extracts (HDM body extract) derived from *Dermatophagoides pteronyssinus* (*D. pteronyssinus*) were obtained from Citeq Biologics (Groningen, The Netherlands, see Table 1). The HDM extracts are produced using borate buffer as a primary buffer. No de-fatting procedure has been applied. The indicated concentrations refer to the total protein content as quantified by BCA assay (ThermoFisher, Rockford, US). Purified natural Der p 1 (nDer p 1) and Der p 2 (nDer p 2) were acquired from Inbio (Charlottesville, VA, United States) and Citeq Biologics, respectively.

**Table 1:** Overview of HDM extracts and purified HDM allergens.

HDM product	Manufacturer	Batch	Protein content [mg/g; BCA]	Allergen content [mg/g; ELISA]	Proteolytic activity	Endotoxin content [EU/g]
*D. pteronyssinus extract*	Citeq Biologics	15G10	325	Der p 1: 41.25 (12.7%) Der p 2: 8.3 (2.6%)	Cysteine protease: 5185 U/mg Serine protease: Trypsin: 437 mU/mg Chymotrypsin: 12.3 mU/mg Elastase: no activity	4.6 ×10^6^
		20B12	369	Der p 1: 42.5 (11.5%) Der p 2: 2.65 (0.7%)	Cysteine protease: 6100 U/mg Serine protease: Trypsin: 347 mU/mg Chymotrypsin: 2.7 mU/mg Elastase: 0.01 mU/mg	195–5.5 ×10^3^
		20A05	384	Der p 1: 41.0 (10.7%) Der p 2: 3.7 (1.0%)	Cysteine protease: 5185 U/mg Serine protease: Trypsin: 437 mU/mg Chymotrypsin: 12.3 mU/mg Elastase: no activity	1.1–5.4 ×10^3^
*D. pteronyssinus* mite body extract	Citeq Biologics	18H09	428	Der p 1: 5.5 (1.3%) Der p 2: 3.3 (0.8%)	Cysteine protease: 5687 U/mg Serine protease: 917 μU/mg	1.5 ×10^4^
nDer p 1	Citeq Biologics	17C14			Cysteine protease: 5270 U/mg	70
	Inbio	6,001,814			>160 RFU @ 0.25 μg/mL	≤ 10
nDer p 2	Citeq Biologics	20B01				5.3 ×10^3^
	Inbio	29,090				≤ 30

*D. pteronyssinus* whole culture extracts are produced by extracting the soluble compounds derived from the mite bodies, together with the mite’s excrements and residual culture medium. Due to the inclusion of fecal particles in whole culture extract, secreted allergens, such as the digestive tract enzyme Der p 1, are present in high concentrations. In contrast, HDM body extract is produced by separating the mite bodies from the culture followed by the extraction of mite body-associated soluble compounds. Accordingly, mite body extracts contain lower levels of Der p 1, while the concentration of body-associated Der p 2 is similar in both extract formulations.

### Primary cells

This study was performed according to the Amsterdam University Medical Centers Medical Ethics Committee guidelines in accordance with the Declaration of Helsinki. We used buffy coats of blood bank donors (Sanquin Bloodbank, Amsterdam, The Netherlands) to isolate CD14+ monocytes to generate suspension-growing monocyte-derived DCs (moDCs) as described previously (15). After 6 days of culture in RPMI 1640 (Gibco/Life Technologies, New York, United States) supplemented with 10% fetal calf serum, L-glutamine (2 mM, Lonza, Verviers, Belgium), pen/strep (100 U/mL and 0.1 mg/mL, respectively; Gibco/Life Technologies), interleukin 4 (IL-4, 25 pg./mL, Gibco) and GM-CSF (20 pg./mL, Gibco/Life Technologies) at 37°C and 5% CO_2_ atmosphere, the monocyte population has been differentiating into immature moDCs. We then treated the immature moDCs with medium (−), nDer p 1, nDer p 2, and (modified) HDM extracts in concentrations ranging from 1 to 50 μg/mL, or, as positive control with 10 ng/mL lipopolysaccharides (LPS) derived from *Salmonella typhosa* (Sigma-Aldrich, St. Louis, MO, United States, #L7895-1MG). All stimulations were carried out for 24 h in the presence of 10% FCS.

To inhibit the Syk signaling pathway, we pre-incubated immature moDCs with 1 μM of R406 (Selleck Chemicals, Planegg, Germany), the active metabolite of Fostamatinib, or as a control with the equimolar concentration of its solvent DMSO for 1 h at 37°C prior to stimulation.

To block receptor-induced signaling, we pre-incubated immature moDCs for 1 h at 37°C with 30 μg/mL of TLR4/MD-2-blocking antibody (clone 7E3, Hycult Biotech, Uden, The Netherlands) or 20 μg/mL of antibodies blocking Dectin-1, Dectin-2, or DC-SIGN, respectively, and matched concentrations of mouse IgG1/IgG2a control antibody (Invivogen and Hycult Biotech) prior to stimulation.

### Cell lines

Human embryonic kidney cells (HEK-293, ATCC CRL-1573) and HEK-293 cells stably transfected with complementary DNA (cDNA) coding for TLR4, MD-2, and CD14 HEK-293/TLR4/MD-2/CD14, that were obtained as a kind gift from D. T. Golenbock (16), were cultured in Dulbecco’s Modified Eagle’s Medium (Lonza) at 37°C and 5% CO_2_. After seeding the cultures into 96-well flat-bottom culture plates (Corning) and letting them adhere for 24 h, the cells were treated for 1 h with 30 μg/mL of either anti-human TLR4/MD-2 (clone 7E3, Hycult Biotech) or mouse IgG1 control antibody (Invivogen) prior to stimulation with 10 μg/mL HDM extract or LPS as a positive control.

### Allergen depletion from HDM extract

In order to remove the major allergens Der p 1 and Der p 2, respectively, from HDM extract, 2.5 mg anti-Der p 1 or 2 monoclonal antibody (17) was covalently coupled to 150 mg CNBr-activated Sepharose beads (GE Healthcare, Chicago, United States) as described previously (18). The resulting 1.3 mL anti-Der p 1 or 2-Sepharose (30 mg/mL) was incubated together with 0.25 mg HDM extract overnight on a shaker platform at 4°C. As a control, 1.3 mL of glycine-Sepharose (30 mg/mL) was incubated likewise. Subsequently, the suspensions were centrifuged and the supernatant containing the depleted extracts or control extracts, respectively, were collected. Depletion efficacy was analyzed by ELISA.

### Protein removal from HDM extract

To digest protein in HDM extract, 1 mL HDM extract (0.86 mg/mL) was incubated with 150 μL *Tritirachium album* proteinase K immobilized on agarose beads (1 U, Sigma Aldrich, Darmstadt, Germany) overnight at RT. As controls proteinase K agarose was also incubated with 1 mL PBS, or 1 mL HDM extract was incubated with control agarose. Digestion of the samples was monitored on SDS-PAGE followed by silver staining.

### Endotoxin removal from HDM extract

We have used Pierce™ High Capacity Endotoxin Removal Spin Columns (Thermo Fisher Scientific, Rockford, United States) to reduce the endotoxin content present in HDM extract according to the manufacturer’s instructions for. In short, the spin columns were regenerated with 2 mL 0.2 N NaOH in 95% ethanol for 1.5 h at RT prior to equilibration. Subsequently, HDM extract was added to the spin columns and incubated for 2 h before the endotoxin-low (LowTox) supernatants were collected by centrifugation. The resulting LowTox HDM extract was analyzed by LAL assay (Pierce™ Chromogenic Endotoxin Quant Kit, Thermo Fisher Scientific) and BCA.

### Flow cytometry analysis

We harvested the moDC cultures after 24 h of sample exposure to assess their maturation status by flow cytometry. To that end, we stained for 30 min at 4°C with APC-conjugated mouse anti-CD83 and FITC-conjugated mouse anti-CD86 (all BD Biosciences, New Jersey, United States) diluted in phosphate-buffered saline (PBS) supplemented with 0.5% bovine serum albumin and 0.02% sodium azide. FACS Canto II (BD Biosciences, Franklin Lakes, United States) and FlowJo software v10 were used to perform flow cytometry analysis. Respective values of moDC maturation markers are shown as mean fluorescence intensity (MFI) and presented relative to data obtained from LPS treatment (LPS = 1).

### Cytokine secretion

Likewise, we harvested moDC culture supernatants after 24 h of stimulation in order to quantify secreted cytokines (IL-6, IL-10 and IL-12 p70; Invitrogen respectively) by ELISA. Cytokine data are shown relative to data obtained from LPS treatment (LPS = 1).

### Statistical analysis

For statistical analysis we used GraphPad Prism version 8 (GraphPad Software LLC, San Diego, CA, United States). Statistical analysis was performed either by Student’s *t*-test, ordinary one-way ANOVA followed by Dunnett’s post-hoc analysis test, or repeated-measures one-way ANOVA followed by Tukey’s post-hoc analysis test. *p* ≥ 0.05 was considered not significant (ns); *p < 0.05, **p < 0.01, ***p < 0.001, and ****p < 0.0001.

## Results

### Der p 1 and Der p 2 do not contribute to DCs activation

**Figure 1:**
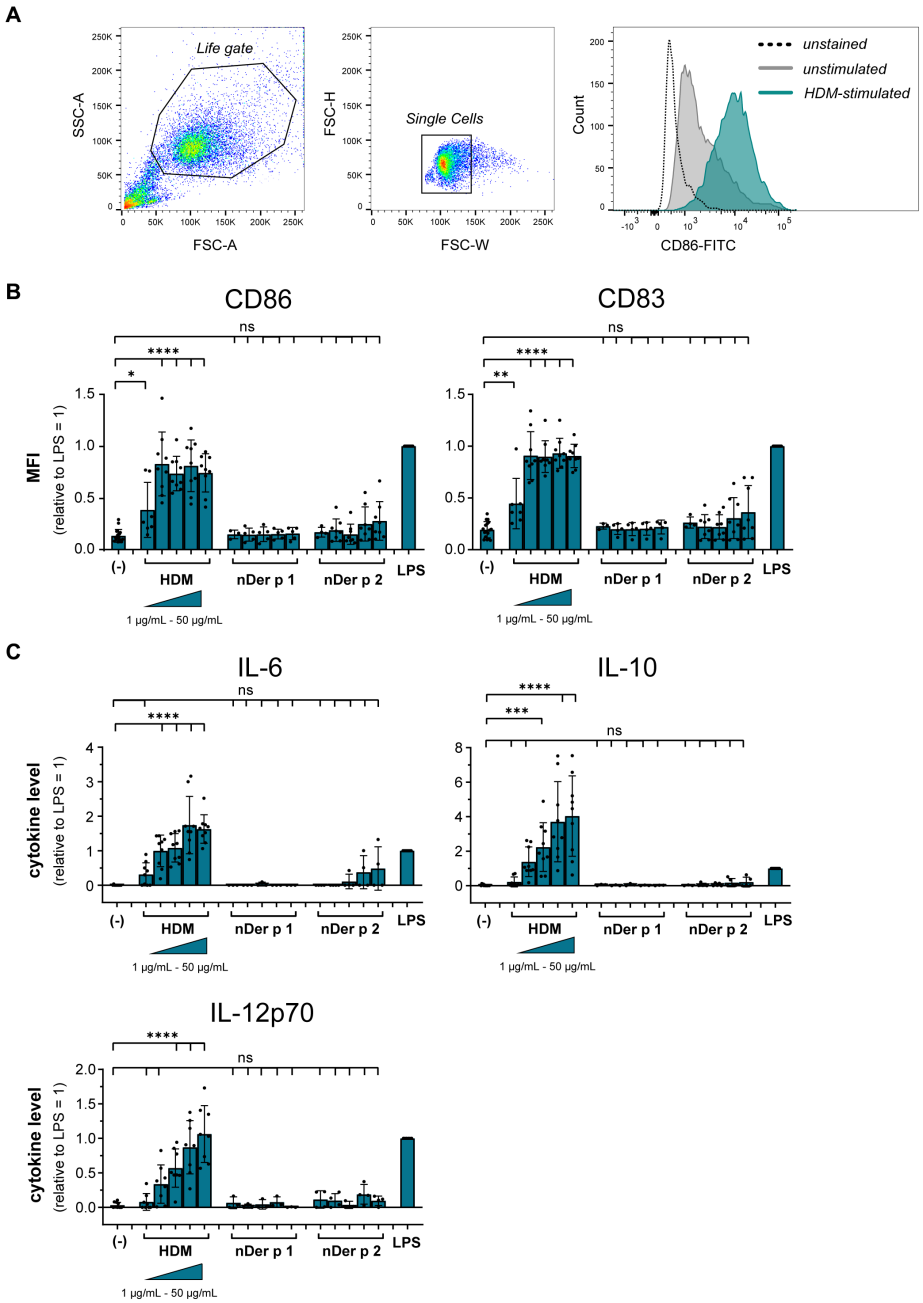
HDM extract, but not the major purified HDM allergens potently activate moDCs. MoDCs were treated for 24 h with 10 ng/mL LPS, 1–50 μg/mL of HDM extract or the natural purified HDM allergens nDer p 1 or nDer p 2. MoDC maturation was assessed by measuring CD86 and CD83 by flow cytometry. **(A)** Gating strategy and representative histograms of an unstained, unstimulated, and HDM extract-stimulated sample. **(B)** Mean fluorescent intensity (MFI) data of the samples are shown relative to levels obtained from cells stimulated for 24 h with 10 ng/mL LPS. The data are presented as the mean ± SD (*n* = 3–10 donors). **(C)** 24 h post-stimulation supernatants were collected and screened for the cytokines IL-6, IL-10, and IL-12p70 by ELISA. The measured data are shown relative to levels obtained from cells stimulated for 24 h with 10 ng/mL LPS. The data are presented as the mean ± SD (*n* = 3–8 donors). ns *p* > 0.05, *p < 0.05, **p < 0.01, ***p < 0.001, ****p < 0.0001 relative to unstimulated cells (one-way ANOVA with Dunnett’s post-hoc analysis test).

**Figure 2:**
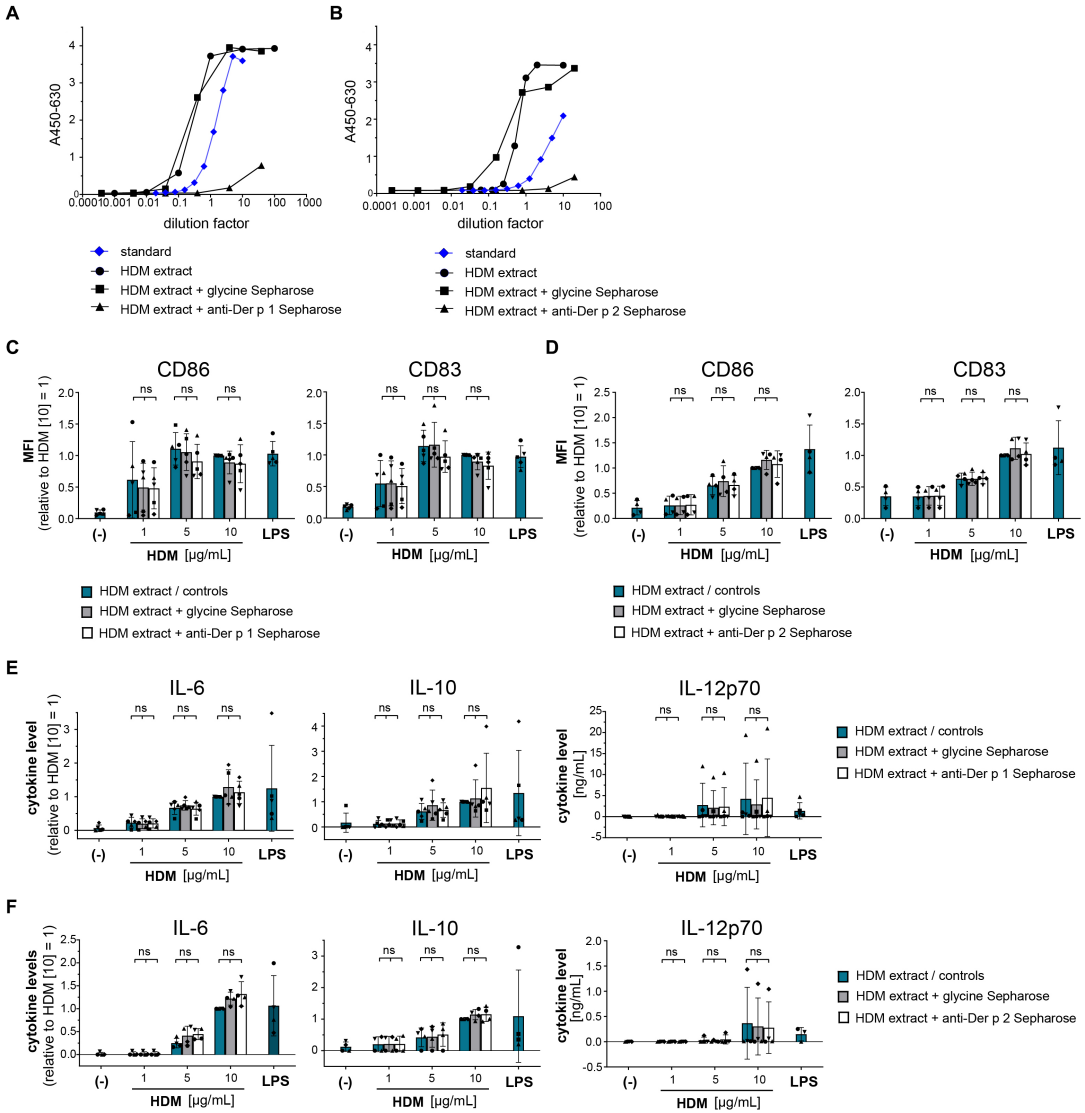
Der p 1 and Der p 2 are not major co-factors in the HDM extract-induced moDC activation. Der p 1 and Der p 2 were depleted from HDM extract by immunoprecipitation with anti-Der p 1/2 Sepharose beads and the depleted HDM extracts were used to stimulate moDCs for 24 h. ELISA analysis of **(A)** Der p 1-depleted and **(B)** Der p 2-depleted HDM extract. **(C,D)** MoDC maturation was assessed by measuring CD86 and CD83 levels by flow cytometry. Mean fluorescent intensity (MFI) data of the samples are shown relative to samples stimulated with 10 μg/mL of native HDM extract. The data are presented as the mean ± SD (*n* = 4–5 donors). **(E,F)** 24 h post-stimulation supernatants were collected and screened for the cytokines IL-6, IL-10, and IL-12p70 by ELISA. The measured data are shown relative to levels obtained from cells stimulated for 24 h with 10 μg/mL of native HDM extract. If undetected cytokine levels did not allow for relative data presentation (IL-12p70), raw data (ng/mL) are shown. The data are presented as the mean ± SD (*n* = 4–5 donors). ns *p* > 0.05, *p < 0.05, **p < 0.01, ***p < 0.001, ****p < 0.0001 (RM-ANOVA with Tukey’s multiple comparison test).

To understand the role of the major HDM allergens Der p 1 and Der p 2 in the early events of sensitization, we treated human moDCs with the purified allergens and HDM extract and determined moDC maturation. Neither nDer p 1 nor nDer p 2, in concentrations up to 50 μg/mL, induced a significant upregulation of the moDC maturation markers CD86 and CD83 (Figures 1A,B; Supplementary Figure S1). In contrast, moDC stimulation with HDM extract resulted in a significant induction of CD86 and CD83. Likewise, we only observed a potent induction of the cytokines IL-6, IL-10, and IL-12p70 only for moDC cultures that were stimulated with HDM extract (Figure 1C), with average cytokine levels of 483.7 ± 129.3 ng/mL, 40.7 ± 34.8 ng/mL, and 1.0 ± 0.8 ng/mL being measured at highest HDM extract concentration for IL-6, IL-10, and IL-12p70, respectively. Especially under the consideration that the proportion of Der p 1 or Der p 2 within the natural extract is restricted to <15% of total protein (Table 1), this further supports no function for the purified major HDM allergens Der p 1 and Der p 2 in HDM-induced DC activation. To investigate the potential of Der p 1 and Der p 2 to induce moDC activation in co-stimulation with other compounds co-delivered upon HDM extract exposure, we immunoadsorbed Der p 1 and Der p 2 from HDM extract, resulting in a ≥ 100-fold reduction of allergen content, respectively (Figures 2A,B). Next we investigated whether the depletion of Der p 1 or Der p 2 affected HDM-induced DC activation. Our data show that depletion of both allergens did not affect the expression levels of DC maturation markers (Figures 2C,D) or cytokine induction (Figures 2E,F). Notably, Der p 1 contributes to the cysteine protease activity present in HDM extract (Table 1). By depleting Der p 1, we conclude to have reduced the cysteine protease reactivity levels present in HDM extract accordingly. Taken together, these data suggest that the biological function of the major HDM allergens Der p 1 and Der p 2 does not directly contribute to DC activation during allergen encounter.

### Proteins in HDM extract are not involved in DC activation

**Figure 3:**
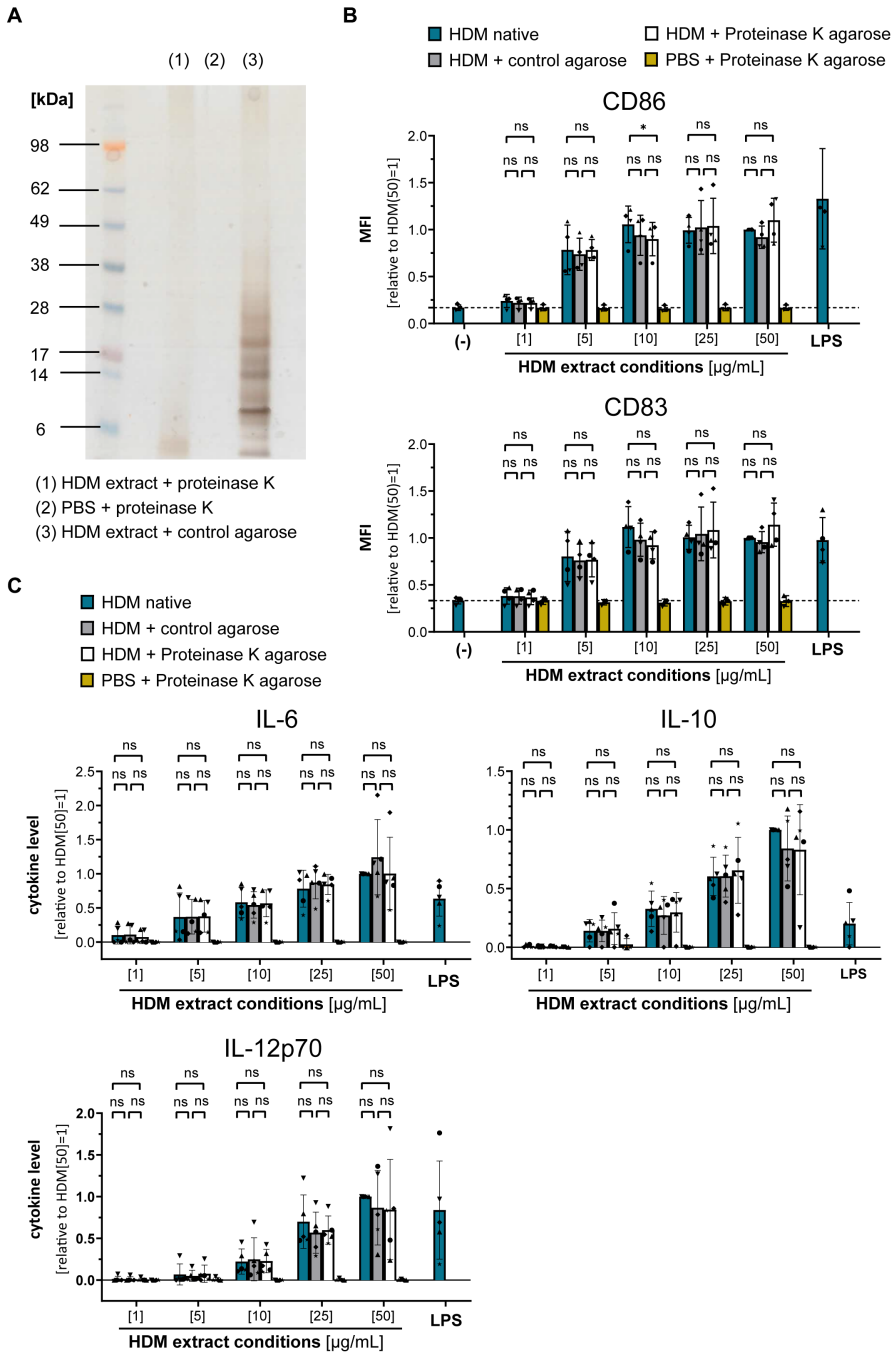
Proteins are not involved in the HDM extract-induced moDC activation. HDM extract was treated with proteinase K and the respective extracts were used to stimulate moDCs for 24 h. **(A)** SDS-PAGE analysis of proteinase K-digested HDM extract. **(B)** MoDC maturation was assessed by measuring CD86 and CD83 levels by flow cytometry. Mean fluorescent intensity (MFI) data of the samples are shown relative to samples stimulated with 50 μg/mL of native HDM extract. The data are presented as the mean ± SD (*n* = 3–4 donors). **(C)** 24 h post-stimulation supernatants were collected and screened for the cytokines IL-6, IL-10, and IL-12p70 by ELISA. The measured data are shown relative to levels obtained from cells stimulated for 24 h with 50 μg/mL of native HDM extract. The data are presented as the mean ± SD (*n* = 4–5 donors). ns *p* > 0.05, *p < 0.05, **p < 0.01, ***p < 0.001, ****p < 0.0001 (RM-ANOVA with Tukey’s multiple comparison test).

To gain further insights into the origin of the immune-activating structures within HDM extract, we investigated extract heterogeneity. While the composition of HDM body extract is limited to soluble compounds derived from the mite bodies, whole culture extracts furthermore contain the mite’s excrements and residual culture medium. Both extract sources induced similar levels of moDC activation as measured by costimulatory molecule expression and cytokine production (Supplementary Figure S2). To investigate the influence of the entire HDM protein repertoire on HDM immune activation via DCs, we incubated HDM whole culture extract with proteinase K to digest all proteins (Figure 3A). Strikingly, total protein digestion neither reduced the level of moDC maturation (Figure 3B) nor the resulting cytokine response (Figure 3C). This further supports that neither Der p 1, Der p 2, nor other protein components are directly involved in activating DCs during sensitization.

### Syk and TLR4 are involved in HDM-induced cytokine response by DCs

**Figure 4:**
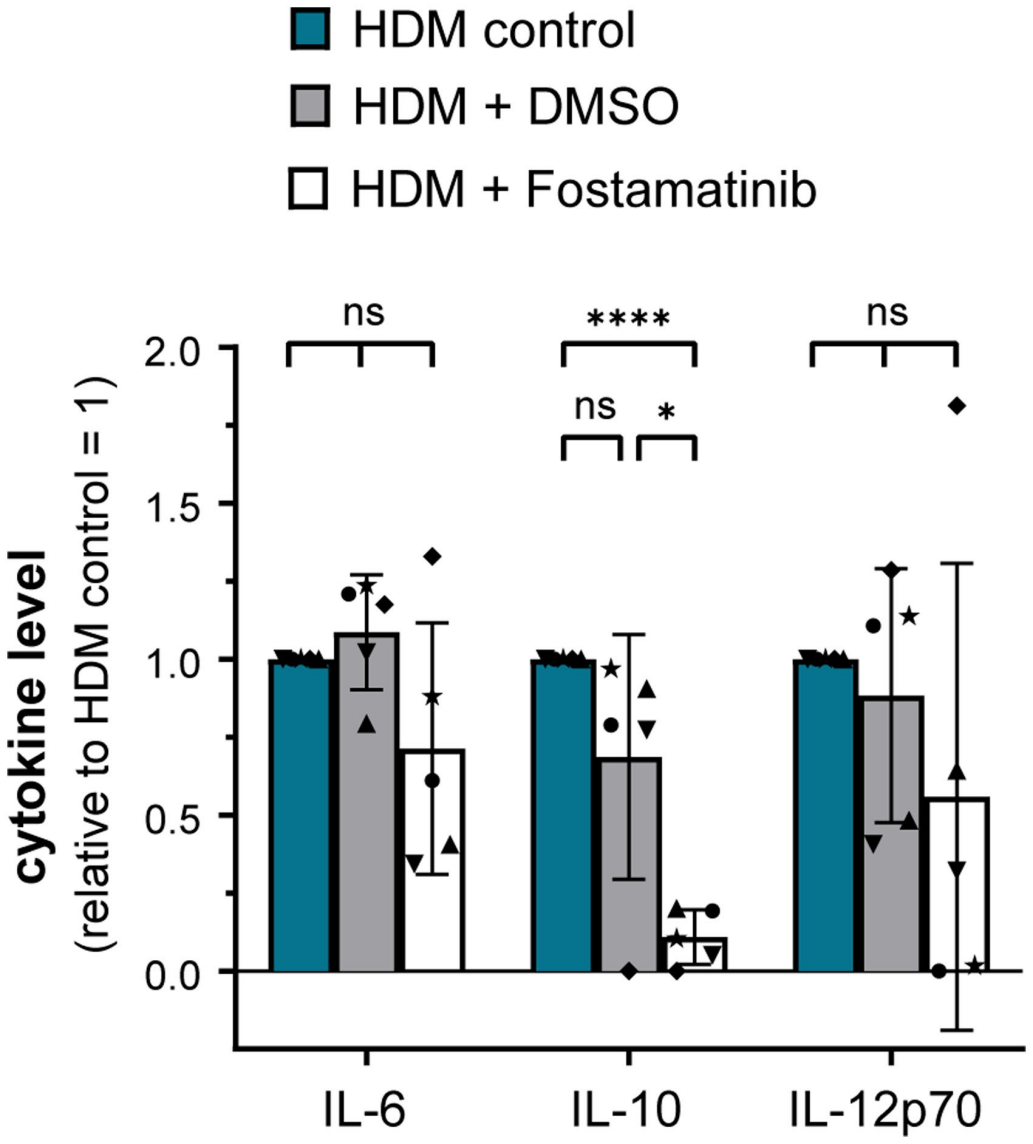
Syk signaling pathway is involved in the HDM extract-induced moDC cytokine response. MoDCs were incubated for 1 h at 37°C with either Fostamatinib or equimolar concentration of its solvent DMSO prior to addition of 5 μg/mL HDM extract. After 24 h of stimulation, the culture supernatants were collected and screened for the cytokines IL-6, IL-10, and IL-12p70 by ELISA. The measured data are shown relative to levels obtained from untreated cells (control group) stimulated for 24 h with HDM extract. The data are presented as the mean ± SD (*n* = 5 donors). ns *p* > 0.05, *p < 0.05, **p < 0.01, ***p < 0.001, ****p < 0.0001 (RM-ANOVA with Tukey’s multiple comparison test).

As proteinase K treatment of HDM extract did not abrogate DC activation, it seems likely that the receptor(s) involved in HDM immune activation recognize non-protein ligand structures. Within the class of pattern recognition receptors (PRRs), C-type lectin receptors (CLRs) and several members of the Toll-like receptor (TLR) family interact with a spectrum of glycans and (glyco)lipid structures (19, 20). We investigated the involvement of the CLRs Dectin-1, Dectin-2, and DC-SIGN in HDM-induced DC activation. Neither antibodies against Dectin-1, Dectin-2 nor DC-SIGN abrogated HDM-induced cytokine response (Supplementary Figure S3). As not only the CLRs Dectin-1 and Dectin-2, but also other receptor types signal via the key signaling molecule Syk (21), we investigated the involvement of Syk in HDM-induced innate immune responses. Notably, Fostamatinib, a pharmacological Syk inhibitor, significantly blocked HDM-induced cytokine responses, especially IL-10 (Figure 4).

**Figure 5:**
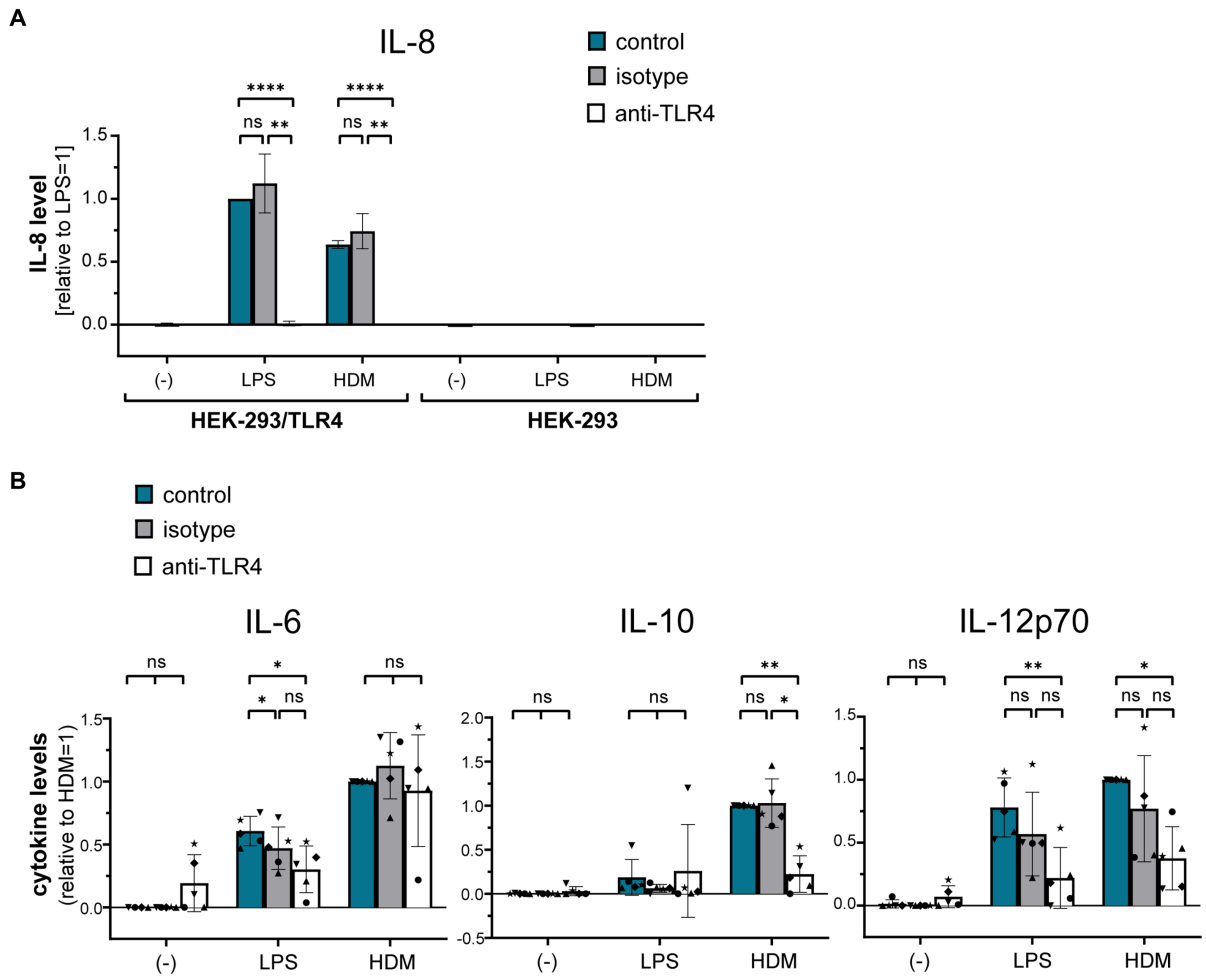
The role of TLR4 in HDM-induced moDC activation. **(A)** A HEK-293/TLR4 reporter cell line and the parental HEK-293 control cell line were incubated for 1 h at 37°C with either TLR4-blocking antibody or the respective isotype control antibody. Subsequently, cultures were exposed for 24 h to 10 ng/mL LPS or 10 μg/mL HDM extract, after which the culture supernatants were collected and screened for by ELISA for IL-8, that is expressed upon TLR4 engagement. The measured data are shown relative to levels obtained from LPS stimulation. The data are presented as the mean ± SD (*n* = 3–4 donors). **(B)** MoDCs were incubated for 1 h at 37°C with either TLR4-blocking antibody or the respective isotype controls and subsequently exposed for 24 h to 10 ng/mL LPS or 50 μg/mL HDM extract. Culture supernatants were collected and screened for the cytokines IL-6, IL-10, and IL-12p70 by ELISA. The measured data are shown relative to levels obtained from untreated (control group) cells stimulated with HDM extract. The data are presented as the mean ± SD (*n* = 5 donors). ns *p* > 0.05, *p < 0.05, **p < 0.01, ***p < 0.001, ****p < 0.0001 (RM-ANOVA analysis with Tukey’s multiple comparison test).

**Figure 6:**
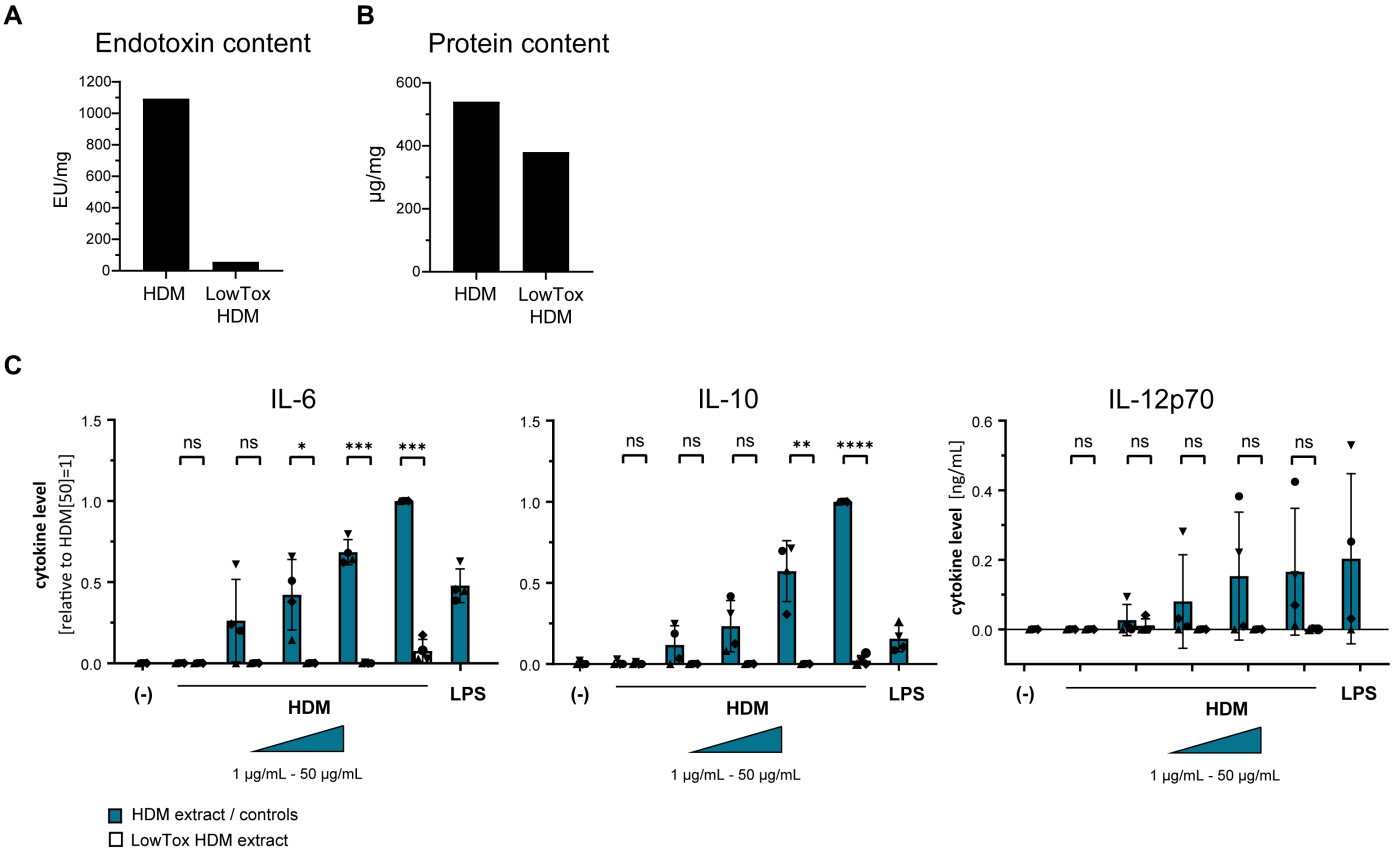
The role of endotoxins in HDM-induced moDC activation. Endotoxins were depleted from HDM extract and the resulting LowTox HDM extract was analyzed for **(A)** removal capacity and **(B)** selectivity. **(C)** Native HDM extract and LowTox HDM extract were used to treat moDCs. After 24 h, culture supernatants were collected and screened for the cytokines IL-6, IL-10, and IL-12p70 by ELISA. The measured data are shown relative to levels obtained from cells stimulated with 50 μg/mL HDM extract. If undetected cytokine levels did not allow for relative data presentation (IL-12p70), raw data (ng/mL) are shown. The data are presented as the mean ± SD (*n* = 4 donors). ns *p* > 0.05, *p < 0.05, **p < 0.01, ***p < 0.001, ****p < 0.0001 (paired *t*-test).

Using a TLR4-HEK-293 reporter cell line, we furthermore investigated whether HDM extract activates TLR4. Our data show that HDM extract induced IL-8 in the TLR4-reporter cell line, which was abrogated when the reporter cell line was pre-incubated with a TLR4-blocking antibody (Figure 5A). This demonstrates that HDM extract contains TLR4 agonists. Blocking TLR4 on moDCs modified the induced cytokine profile by significantly reducing the induced IL-10 response and showed a trend toward reduced IL-12p70 levels, whereas IL-6 levels were unaffected (Figure 5B). Endotoxins derived from bacterial cell walls are well-studied ligands for TLR4 (16). To investigate the involvement of endotoxins in the induction of HDM-directed immune responses, we reduced 95% of the endotoxins present in HDM extract (Figure 6A). The generated LowTox HDM extract showed a 30% reduction in total protein content (Figure 6B), which might be a result of protein-endotoxin aggregation leading to reduced sample recovery. When treating moDCs with native and LowTox extract, we observed a strong decrease of the induced cytokine responses (Figure 6C).

Taken together, these data highlight the role of endotoxins and TLR4 in the activation of DCs toward HDM and might suggest that there is crosstalk between Syk and TLR4 signaling upon HDM interactions.

## Discussion

The role of HDM allergens as major IgE-reactive structures has been studied intensively and let to the identification of several protein classes of various biological activities (3). However, the events initiating HDM-directed adaptive responses remain unclear. We here focused on the effect of HDM on DCs as orchestrators of T and B cell responses. Our data suggest that protein components including the major HDM allergens Der p 1 and Der p 2 are not directly involved in DC activation. Interestingly, we found that both TLR4, that is widely expressed on DCs, and Syk signaling are involved in the HDM-induced cytokine responses.

The fact that the major IgE-reactive structures are not involved in the initial events of triggering self-directed adaptive immune responses might appear surprising. However, our data strongly suggest that neither purified Der p 1 nor Der p 2 induce DC activation by themselves and likewise do not possess immunomodulatory properties within the cognate extract environment. The absence of an intrinsic stimulatory effect of Der p 1 and Der p 2 on DCs becomes even clearer if we take into account that the purified allergens were used in a highly concentrated form compared to their concentration within HDM extract. Data from mouse experiments comparing the effect of the purified peanut allergens Ara h 1, Ara h 2, and Ara h 6 in comparison with peanut extract report poor immune-activating properties for the purified peanut allergens (6). Also other airborne allergens confirm poor intrinsic immunogenicity. Aglas et al. demonstrated that the major birch pollen allergen Bet v 1 is not the sensitization-driving force and Th2 polarization upon immunization with birch pollen extract occurs independently of Bet v 1 (5). Our data align with these observations, suggesting that additional immune interactions are needed in HDM allergy to trigger DC activation. Nevertheless, recent work illustrated the complexity of cellular crosstalk taking place at and beyond the mucosal barrier including an interplay of ECs, APCs, and innate lymphoid cells in priming adaptive responses (7, 22). We hence cannot exclude that purified HDM allergens have immunogenic effects on other cell types than DCs, which might in turn deliver activating signals to DCs.

The digestion of the entire protein content present in HDM extract indicated that the structures responsible for DC activation are less likely to be protein-based, which draws attention to the presence of adjuvant components originating from the allergen source. HDM extract is a complex mix of structures combining-depending on its source-not only multitudes of mite body-derived structures, such as proteins, carbohydrates, and (glyco-)lipids, but also residual culture medium and mite excrements. The sensitizing potential of HDM might hence be explained by the synergistic effect of adjuvant structures originating from within the mite body and the mite environment. As potent APCs, DCs express a broad array of PRRs, of which TLRs and CLRs recognize wide groups of pathogen-associated molecular patterns (PAMPs) to tailor respective protective responses. Based on specific glycan PAMPs, the CLRs Dectin-1 and Dectin-2 are major players in anti-fungal responses (23, 24) and might therefore also be of relevance for HDM-induced immune reactions. However, our data show an involvement of the innate immune receptor TLR4 in the HDM extract-induced IL-10 and IL-12 responses, which are critical for T cell polarization (25–27). Earlier mouse studies have highlighted a potential auto-adjuvant property of Der p 2 due to its structural and functional homology with MD-2, the LPS-binding protein of the TLR4 signaling cascade (14). Our observations based on purified Der p 2 and Der p 2-depleted HDM extract do, however, not provide support for auto-adjuvant properties to activate or modify DC reactivity. While further *in-vivo* studies have confirmed the involvement of TLR4 in the induction of HDM-mediated asthma, the respective studies furthermore emphasized the effects on ECs in this process (28–30). A central role of the TLR4 pathway in DC activation was furthermore reported for birch pollen extract, where the authors identified the TLR4 ligand within the lipid fraction of the allergen extract (31). While several structures, such as (O-linked) mannans found in the cell wall of yeasts (32, 33), glycosaminoglycans (34), and even metal ions (35) have been linked to inducing immunomodulatory responses via TLR4, LPS as an endotoxin derived from the cell wall of gram-negative bacteria is known as the classical ligand that induces a pro-inflammatory response via TLR4 and its co-receptors MD-2 and CD14 (36, 37). Given the natural heterogeneity of HDM extract, the presence of endotoxins herein is undeniable and our data confirm a central role of endotoxins in the induction of DC activation. Previous work has emphasized the impact of LPS dosage as a critical parameter for Th1/2 polarization (38). Besides the dosage also the origin of those endotoxins present in HDM extract might be of interest. In this context, the mite-inhabiting microbiome shifts into focus. A recent transcriptomic analysis study analyzed the effect of extracts originating from different mite species on airway ECs (39). The authors revealed that HDM extracts derived from *D. farinae* triggered high levels of anti-bacterial immune responses, while fungi were predominantly associated with *D. pteronyssinus*. A recent study deciphering the internal and environmental microbiome of HDM found a dominance of Gram-positive bacteria (40). Hence, the origin and immunomodulatory potency of endotoxins within HDM are a subject worthwhile to explore in the context of allergic sensitization.

Interestingly, we observed overlapping patterns for TLR4 and Syk signaling, with both pathways being mainly involved in the HDM-induced IL-10 and, to a less clear extent, also IL-12p70 responses. Notably, IL-10 and IL-12 are important factors for Th2 priming. While Syk signaling is among others associated with Dectin-1-and Dectin-2-induced immune responses (41–43), limited information about an involvement of Syk in TLR4 signaling is known so far. However, earlier work sharing our observation has highlighted Syk as a modulator of LPS-induced TLR4 responses in human monocytic cells and let the authors speculate on a ligand-induced conformational change in TLR4 that might cause an activation of pre-associated Syk, allowing it to phosphorylate TLR4 (44).

Taken together, our findings suggest that Der p 1 and Der p 2 as the major targets of adaptive immune responses do not possess intrinsic capacities to activate DCs, but are rather dependent on the immunostimulatory function of non-allergenic entities present within HDM extract. Our finding that TLR4 and Syk signaling showed similar involvements in the induced cytokine signatures might together with knowledge from literature suggest a crosstalk of both signaling pathways for HDM-directed innate immune responses (45–47). However, neither TLR4 nor Syk signaling could abrogate the entire HDM-induced cytokine response, pointing towards the involvement of various receptors synergistically shaping the induced reaction in response to endotoxin trigger. The knowledge about the involvement of TLR4 and Syk signaling in HDM-induced innate immune responses can be harnessed for adjuvant development in allergen immunotherapy for instance by screening for TLR4 agonists that do no elicit Th2 cytokine responses. In this way, immune responses could be activated and reprogrammed, which might allow for the usage of lower allergen concentrations, that in turn favor therapy safety.

## Data availability statement

The raw data will be made available upon request.

## Ethics statement

Ethical review and approval was not required for the study on human participants in accordance with the local legislation and institutional requirements. The patients/participants provided their written informed consent to participate in this study.

## Author contributions

SB and JA designed the involved experiments. SB, JA, and EZ-W conducted the involved experiments. SB wrote the manuscript and created the figures. KG provided the study with HDM extract and purified allergens. RR and TG provided guidance during the entire study and critically read and revised all versions of the manuscript. EJ and ST provided further project guidance and critically read the manuscript to provide valuable additions. All authors contributed to the article and approved the submitted version.

## Funding

This project is embedded in the LSH-TKI project DC4Balance, which was supported by Health Holland (LSHM18056-SGF).

## Conflict of interest

RR received consulting fees and/or speaker’s fees from Angany Inc., HAL Allergy BV, and Citeq BV, ThermoFisher Scientific Reacta Healthcare Ltd., Mission MightyMe, AB Enzymes and ALK Abello. KG is the CEO/owner of Citeq Biologics.

The remaining authors declare that the research was conducted in the absence of any commercial or financial relationships that could be construed as a potential conflict of interest.

## Publisher’s note

All claims expressed in this article are solely those of the authors and do not necessarily represent those of their affiliated organizations, or those of the publisher, the editors and the reviewers. Any product that may be evaluated in this article, or claim that may be made by its manufacturer, is not guaranteed or endorsed by the publisher.
