# Supplementary material for: Toll-like receptor 4 and Syk kinase shape dendritic cell-induced immune activation to major house dust mite allergens
Source: Front Med (Lausanne). 2023 Aug 8;10:1105538. doi: 10.3389/fmed.2023.1105538 (PMC10442820; doi:10.3389/fmed.2023.1105538)
Supplement: Supplementary file 1 [file Data_Sheet_1.PDF]

Supplementary Material

Toll-like receptor 4 and Syk kinase shape dendritic cell-induced immune activation to major house dust mite allergens.

Stefanie Busold[1, 2], Jaap H. Akkerdaas[1, 2], Esther M. Zijlstra-Willems[1, 2], Kees van der Graaf[3], Sander W. Tas[1, 2, 4], Esther C. de Jong[1, 2], Ronald van Ree[1, 2, 5, *], Teunis B. H. Geijtenbeek[1, 2]

*** Correspondence:** Corresponding Author: t.b.geijtenbeek@amsterdaumc.nl

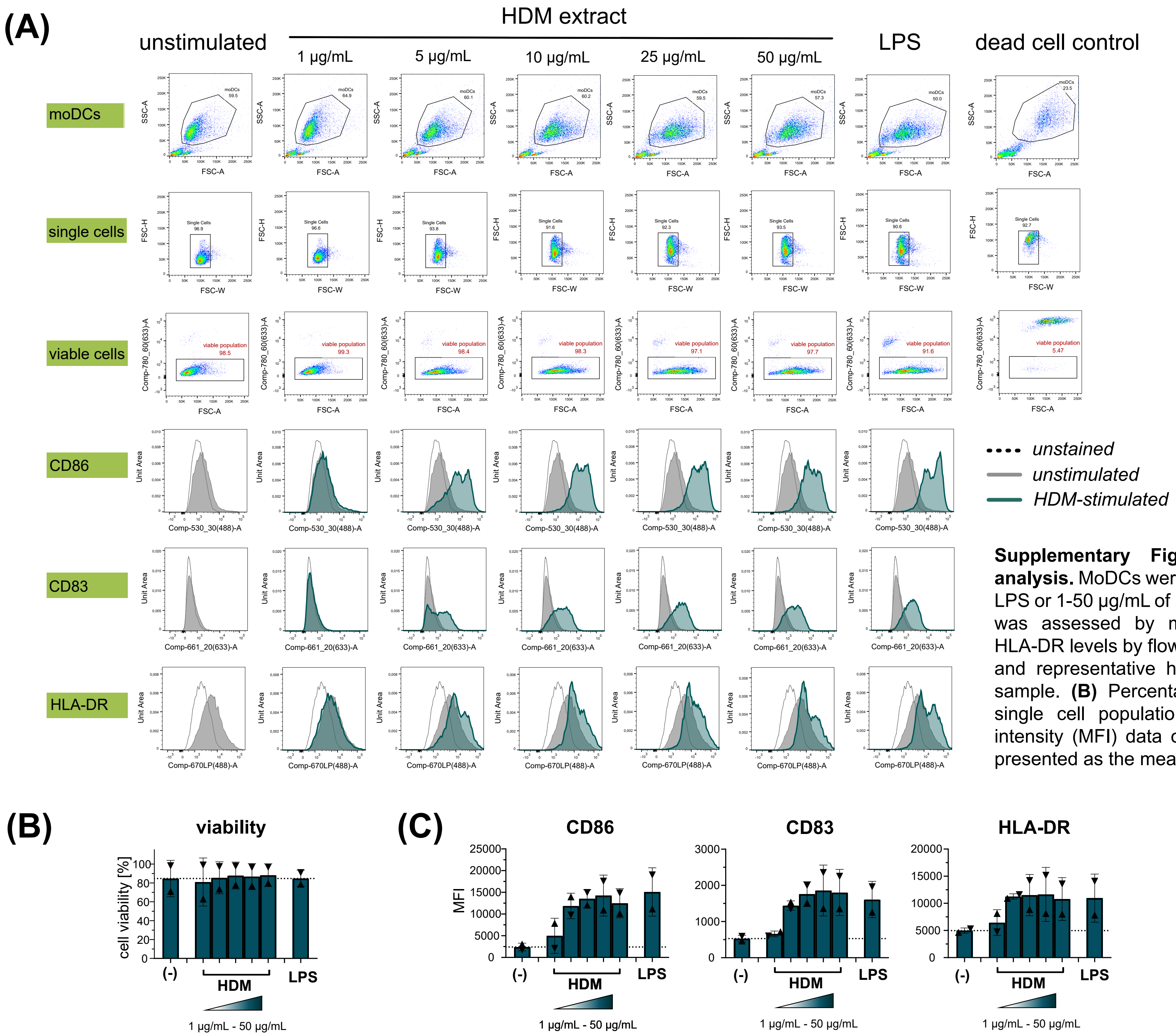

Supplementary Figure 1. MoDC maturation analysis. MoDCs were treated for 24 h with 10 ng/mL LPS or 1-50 µg/mL of HDM extract. MoDC maturation was assessed by measuring CD86, CD83, and HLA-DR levels by flow cytometry. **(A)** Gating strategy and representative histograms of a representative sample. **(B)** Percentage of viable cells within the single cell population and **(C)** mean fluorescent intensity (MFI) data of the samples. The data are presented as the mean ± SD (n=2 donors).

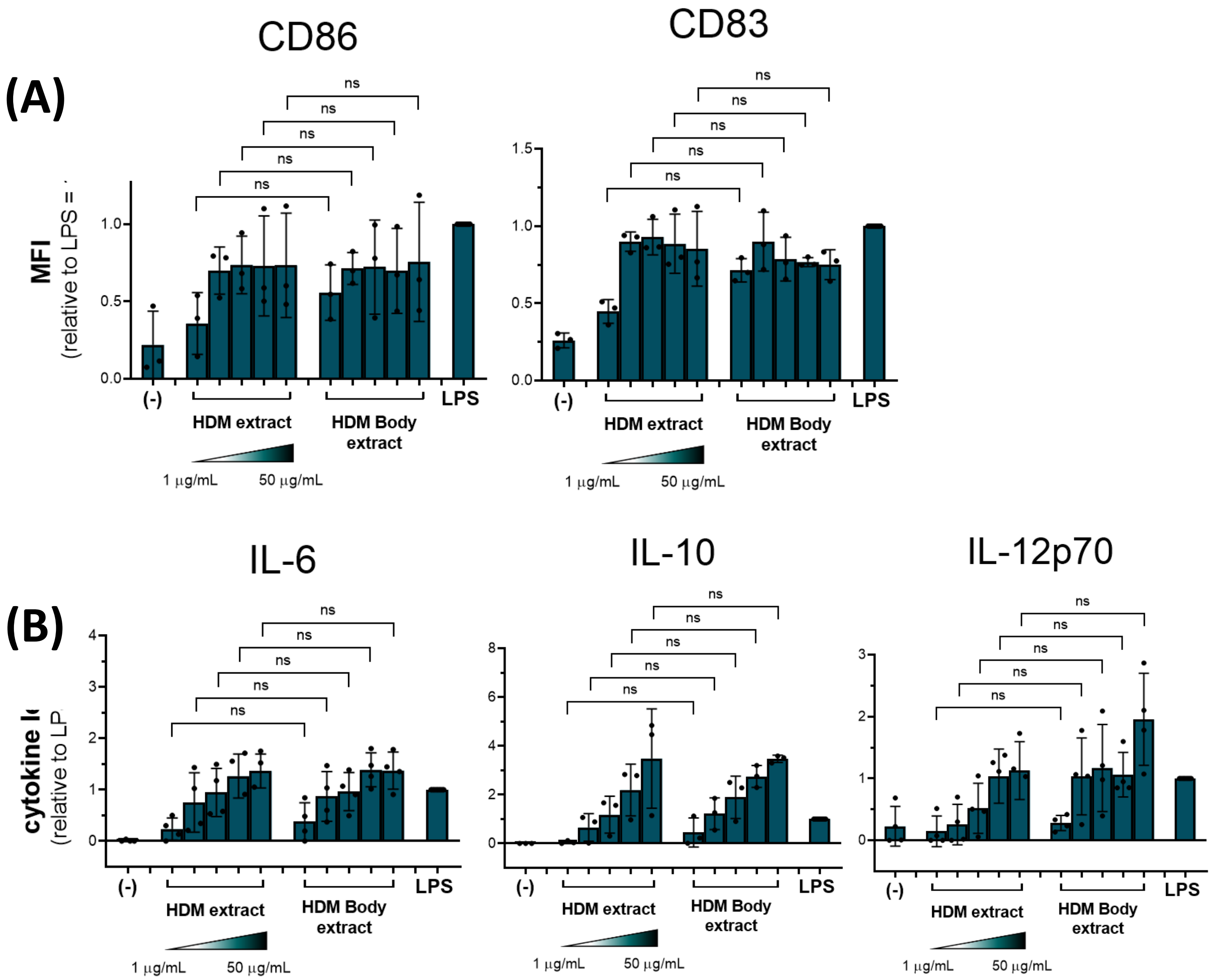

Supplementary Figure 2. HDM whole culture extract and HDM body extract both potently activate moDCs. MoDCs were treated for 24 h with 10 ng/mL LPS or 1-50 µg/mL of HDM extract derived either from whole mite culture or mite bodies. **(A)** MoDC maturation was assessed by flow cytometry. Mean fluorescent intensity (MFI) data of the samples are shown relative to levels obtained from cells stimulated with 10 ng/mL LPS. **(B)** Culture supernatants were collected and screened for the cytokines IL-6, IL-10, and IL-12p70 by ELISA. The measured data are shown relative to levels obtained from cells stimulated with 10 ng/mL LPS.

The data are presented as the mean ± SD (n=3-4 donors).

n.s. $p > 0.05$, * $p < 0.05$, ** $p < 0.01$, *** $p < 0.001$, **** $p < 0.0001$ relative to unstimulated cells (RM-ANOVA with Tukey's multiple comparison test).

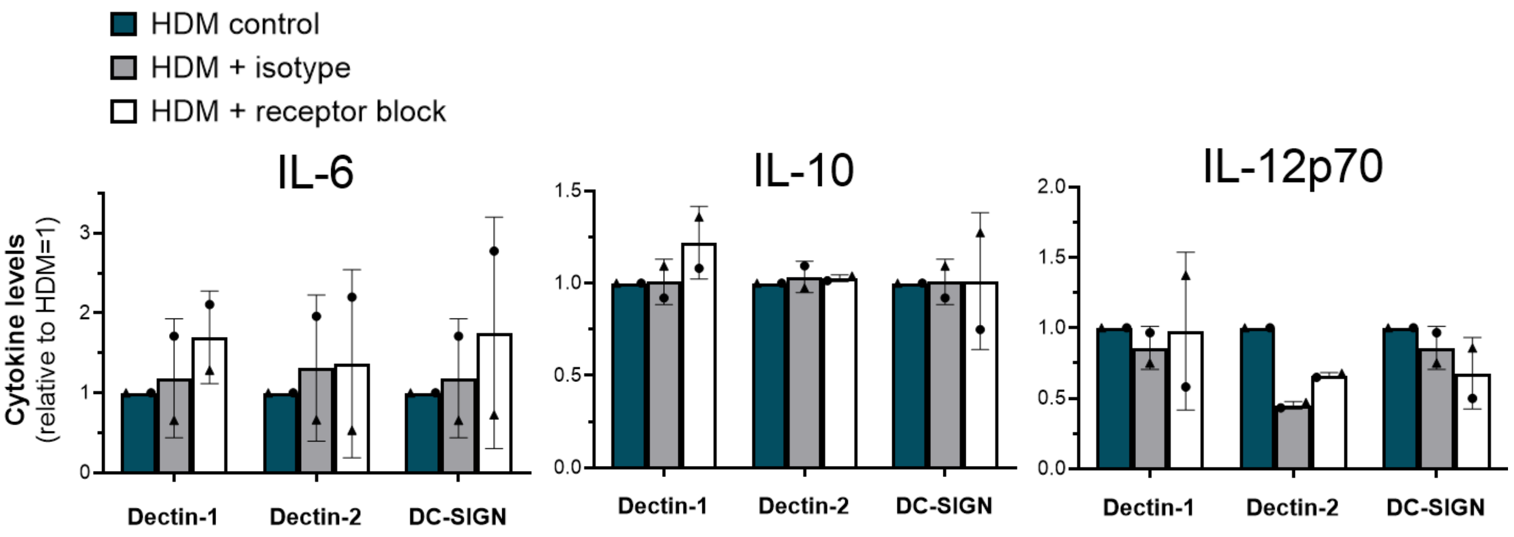

Supplementary Figure 3. The role of CLRs in HDM-induced moDC activation. MoDCs were incubated for 1 h at 37 °C with either Dectin-1-, Dectin-2-, or DC-SIGN-blocking antibody or the respective isotype controls and subsequently exposed for 24 h to 10 ng/mL LPS or 50 µg/mL HDM extract. Culture supernatants were collected and screened for the cytokines IL-6, IL-10, and IL-12p70 by ELISA. The measured data are shown relative to levels obtained from untreated (control group) cells stimulated with HDM extract. The data are presented as the mean ± SD (n=2 donors).
